# Label-free detection of uptake, accumulation, and translocation of diesel exhaust particles in ex vivo perfused human placenta

**DOI:** 10.1186/s12951-021-00886-5

**Published:** 2021-05-17

**Authors:** Eva Bongaerts, Leonie Aengenheister, Battuja B. Dugershaw, Pius Manser, Maarten B. J. Roeffaers, Marcel Ameloot, Tim S. Nawrot, Hannelore Bové, Tina Buerki-Thurnherr

**Affiliations:** 1 Centre for Environmental Sciences, Hasselt University, Agoralaan Building D, 3590 Diepenbeek, Belgium; 2 Laboratory for Particles-Biology Interactions, Empa, Swiss Federal Laboratories for Materials Science and Technology, St. Gallen, Switzerland; 3 Centre for Surface Chemistry and Catalysis, KU Leuven, Leuven, Belgium; 4 Biomedical Research Institute, Hasselt University, Agoralaan Building C, 3590 Diepenbeek, Belgium; 5 Department of Public Health and Primary Care, KU Leuven, Herestraat 49, Box 703, 3000 Leuven, Belgium

**Keywords:** Environmental pollution, Diesel exhaust particles, In utero exposure, Ex vivo placental perfusion, Nanosafety

## Abstract

**Background:**

Pregnant women and developing fetuses comprise a particularly vulnerable population as multiple studies have shown associations between prenatal air pollution exposure and adverse pregnancy outcomes. However, the mechanisms underlying the observed developmental toxicity are mostly unknown, in particular, if pollution particles can cross the human placenta to reach the fetal circulation.

**Results:**

Here, we investigated the accumulation and translocation of diesel exhaust particles (DEPs), as a model particle for combustion-derived pollution, in human perfused placentae using label-free detection by femtosecond pulsed laser illumination. The results do not reveal a significant particle transfer across term placentae within 6 h of perfusion. However, DEPs accumulate in placental tissue, especially in the syncytiotrophoblast layer that mediates a wealth of essential functions to support and maintain a successful pregnancy. Furthermore, DEPs are found in placental macrophages and fetal endothelial cells, showing that some particles can overcome the syncytiotrophoblasts to reach the fetal capillaries. Few particles are also observed inside fetal microvessels.

**Conclusions:**

Overall, we show that DEPs accumulate in key cell types of the placental tissue and can cross the human placenta, although in limited amounts. These findings are crucial for risk assessment and protection of pregnant women and highlight the urgent need for further research on the direct and indirect placenta-mediated developmental toxicity of ambient particulates.

**Figure:**
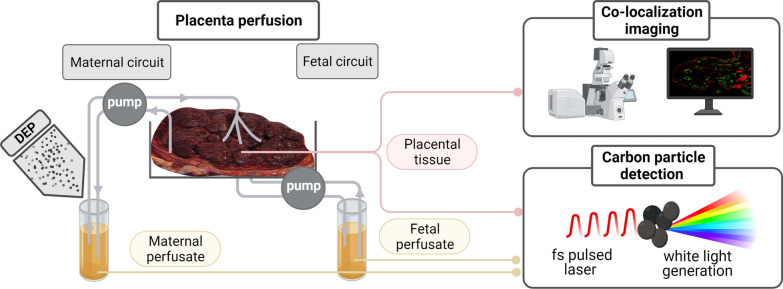

**Supplementary Information:**

The online version contains supplementary material available at 10.1186/s12951-021-00886-5.

## Background

Air pollution exposure, in particular to traffic-related ambient particulate matter, is associated with adverse pregnancy and fetal outcomes like small for gestational age [1, 2], low full-term birth weight [3, 4], or autism spectrum disorders [5]. The underlying causes are still not fully understood, but first studies in humans linked prenatal particulate matter exposure to elevated systemic inflammation levels in the fetus [6], intrauterine inflammation [7], lower placental weight [8], reduction of placental mitochondrial DNA [9] and increased placental nitrosative stress levels [10]. Recently, gestational air pollution has been linked with shorter newborn telomere length, a biomolecular marker in the core axis of aging, both in the placenta and cord blood [11]. Observed adverse effects could be caused by three major pathways: (i) the placental translocation of particles provoking direct fetotoxic effects, and/or (ii) the indirect maternal mediated effects caused by the accumulation of particles in maternal tissues causing maternal organ dysfunction or release of inflammatory mediators and soluble signaling factors that can reach the placenta and fetus and possibly induce toxic effects and/or (iii) the indirect placental mediated effects caused by the accumulation of particles in placental tissues disrupting essential barrier and transport functions or inducing the release of secondary mediators that indirectly interfere with fetal development [12, 13]. In this regard, Bové et al. recently detected black carbon (BC) particles, a constituent of combustion-derived particulate matter, in the placental tissue of pregnant women in function of ambient exposure levels [14]. Although proof of placental translocation to the fetal circulation is still lacking, this study confirmed, in a real-life scenario, that BC particles inhaled by the mother could reach and accumulate in placental tissue and potentially adversely affect its function. A new study strengthened these findings and observed the uptake of inhaled air pollution-derived particles by macrophage-enriched placental cells [15]. However, the authors acknowledge limitations in correlating the uptake to specific placental cell types (e.g., macrophages, trophoblasts, and leukocytes) in intact placental tissues as well as to the origin of these cells (i.e., maternal and fetal cells). Such information is imperative to understand the contribution of direct and/or indirect pathways to the observed developmental toxicity. Therefore, there is still an urgent and unmet need to provide human-relevant data on fetal translocation of ambient combustion-related particulates, including quantitative data on transfer rates and elucidate cell type-specific localization of the particles in the native placental tissue.

**Fig. 1:**
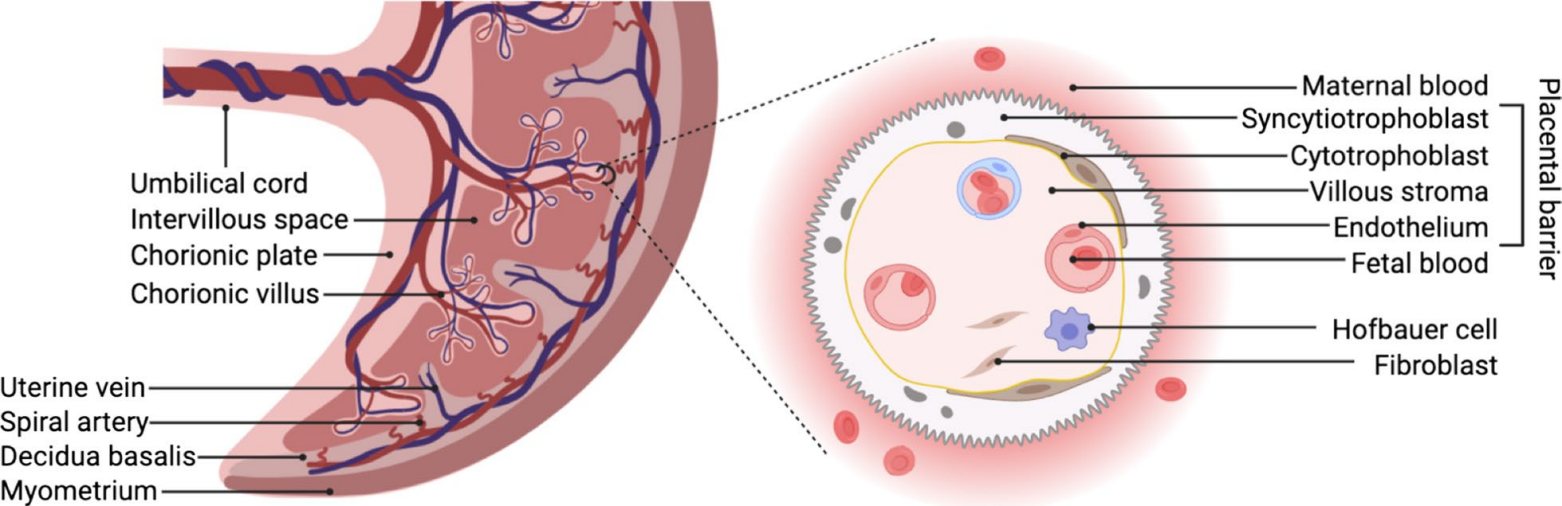
The placental barrier. Schematic representation of the human placental structure in term pregnancy. From the chorionic plate, the umbilical cord and chorionic villi originate. The intervillous space is filled with maternal blood entering via remodeled maternal spiral arteries and leaving via the uterine veins. The syncytiotrophoblast, which is formed by the fusion of cytotrophoblasts, faces the maternal blood and therefore constitutes the first layer to get into contact with systemic DEPs. While a complete cytotrophoblast layer exists in early pregnancy, these cells are sparse at the end of pregnancy. Beneath this syncytium lay the villous stroma containing fibroblasts and placental macrophages (i.e., Hofbauer cells) and the endothelial cell-lined fetal microvessels. Created with BioRender.com

A recent review by Bongaerts et al. summarizes the current though scarce evidence on the maternal–fetal transfer and fetoplacental accumulation of (ultra)fine particles and nanoparticles [16]. Both wood smoke particles [17] and PM_2.5_ (particulate matter ≤ 2.5 µm) [18] have been shown to accumulate in exposed placental first-trimester trophoblast cell lines (i.e., HTR-8/SVneo) at an applied dose of 0.5 µg/mL. The exposure to these combustion-related particulates caused mitochondrial ultrastructural changes, reduced human chorionic gonadotropin (hCG) secretion, and increased levels of the pro-inflammatory cytokine interleukin-6 [17, 18]. The level of hCG during early gestation is critical as it supports, among others, the maintenance of the corpus luteum (secretion of progesterone and estrogens in the ovaries during the first trimester) [19] and the formation of the syncytiotrophoblast layer [20]. Accordingly, disturbance in the tightly regulated hCG levels, as well as increased levels of pro-inflammatory mediators, could increase the risk of adverse pregnancy outcomes [21, 22]. In experimental animals, “nanoparticle-like” black particles were detected by transmission electron microscopy in the maternal circulation, trophoblastic cells, and fetal blood [23] as well as the fetal olfactory tissue [24] following inhalation of diesel exhaust particles (DEPs) by pregnant rabbits (nose-only inhalation; 1 mg/m^3^, 2 h/day, 5 days/week). Gestational DEP exposure resulted in reduced placental function (i.e., decreased placental efficiency, placental blood flow, and fetal vessel volume) and intra-uterine fetal growth retardation (accompanied with reduced insulin-like growth factor 1 fetal plasma levels) [23], as well as altered-olfactory based behavior in pups [24]. The underlying mechanisms are not fully understood and may include both direct (e.g., toxicity to fetal tissues from translocated particles) and indirect (e.g., inflammation or disturbed hormone secretion in maternal or placental tissue) effects [13]. In general, exposure of pregnant animal models can provide valuable insights into the biodistribution of substances or particles in a living organism, including potential placental transfer towards the fetus. Nonetheless, human-relevant models should be favored since the placenta is known to be the most species-specific organ with unique structure, development, and function [25, 26]. In humans, materno-fetal transfer occurs across several cell layers and basement membranes, more specifically a layer of mononucleated cytotrophoblasts and multinucleated syncytiotrophoblasts facing the maternal blood, the villous stroma containing fibroblasts and Hofbauer cells, and the endothelial cells of the fetal capillary walls (Fig. 1).

To address these challenges and close the current knowledge gap, we aimed to (i) unveil the biodistribution of DEPs as a model particle for combustion-derived pollution in human placental tissue and (ii) establish whether these particles can cross the placenta and reach the fetal circulation. To this end, we employed the current gold standard ex vivo placental perfusion model [27] to assess placental transfer in an intact human tissue under physiologically-relevant perfused conditions. Furthermore, DEP concentrations in placental tissue and maternal and fetal perfusates were determined in a label-free manner via femtosecond pulsed laser microscopy allowing the sensitive and specific detection of DEPs in biological matrices [28].

## Results

### DEPs dispersion and characterization

For this study, we used well-characterized standard reference DEPs from the National Institute of Standards and Technology (NIST^®^, SRM^®^1650b), and particle characteristics are summarized in Table 1. After ultra-sonic preparation of DEPs suspensions, particles showed a mean hydrodynamic diameter (SD) of 257 (16) nm (Z-average) in perfusion medium (PM), and the polydispersity index (PDI) was 0.52 (0.06), demonstrating a rather polydisperse particle suspension and potential particle agglomeration (see Additional file 1: Figure S1 for particle size distribution graph). Likewise, transmission electron microscopy (TEM) images of aerosolized DEPs show a heterogeneous mixture of particles of different sizes and morphologies see references [29, 30]). DEPs were endotoxin-free (< 0.5 EU/mL in Chromogenic Limulus amebocyte lysate (LAL) assay), confirming the absence of bacterial contamination, which could induce unexpected inflammatory responses.

**Table 1:** Characterization of DEPs

Characteristics		DEPs
BET: Specific surface area (m^2^/g)^a^		108
Laser diffraction: Hydrodynamic diameter (nm)^a^	In ddH_2_0 with 0.001% Triton	180 (d(0.5)) 330 (d(0.9))
DLS: Hydrodynamic diameter ( nm)/PDI^b^	In PM	257 (16)/0.52 (0.06)
Zeta potential (mV)^b^	In 10% PM	− 29 (1.46)

BET: Brunauer–Emmet–Teller method; DLS: dynamic light scattering; d(0.5): mean particle-size indicating the diameter below which 50% of the volume is present; d(0.9): mean particle-size indicating the diameter below which 90% of the volume is present; PDI: polydispersity index; PM: perfusion medium

^a^According to the manufacturer (https://www-s.nist.gov/srmors/certificates/1650b.pdf)

^b^Experimentally determined parameters of 4.5 µg/mL DEP suspensions: Hydrodynamic diameter represents Z-average mean (SD); Zeta potential represents mean (SD)

### Calculation of DEP exposure concentration from real-life conditions

The applied DEP concentration was calculated to match a real-life exposure scenario. In a previous study, on average, 2.09 × 10^4^ particles per mm^3^ were found in human placental tissue at term from high exposed mothers to an average BC concentration ranging between 1.70 and 2.42 μg/m^3^ during pregnancy [14]. Assuming that DEPs are round spheres with a mean diameter of 330 nm (according to the manufacturer’s Information, d(0.9), https://www-s.nist.gov/srmors/certificates/1650b.pdf), a particle has a volume of 1.88 × 10^–20^ m^3^ and a mass of 3.38 × 10^–14^ g with a density of 1.80 × 10^6^ g/m^3^ [31]. The average volume of a placenta is 500 cm^3^ [32], leading to an estimated deposition of 353 μg of particles per placenta or 17.7 μg particles per cotyledon (20 cotyledons per placenta) during pregnancy. Taking into account the potential loss of particles to the perfusion system, some diffusion of the suspension into the surrounding tissues, and incomplete tissue uptake of the applied particles, a 3× safety margin was included in the calculation resulting in an applied concentration of 0.45 μg/mL (3 × 17.7 µg/120 mL PM) in the maternal circulation for the perfusion studies. A second approach (based on the extrapolation from expected ambient exposure levels and the expected dose in the circulation for a 24 h exposure) providing similar exposure values and a rationale for the applied safety margin can be found in Additional file 1 (Calculation of DEP exposure concentration from real-life conditions).

### Impact of DEPs on trophoblast viability

**Fig. 2:**
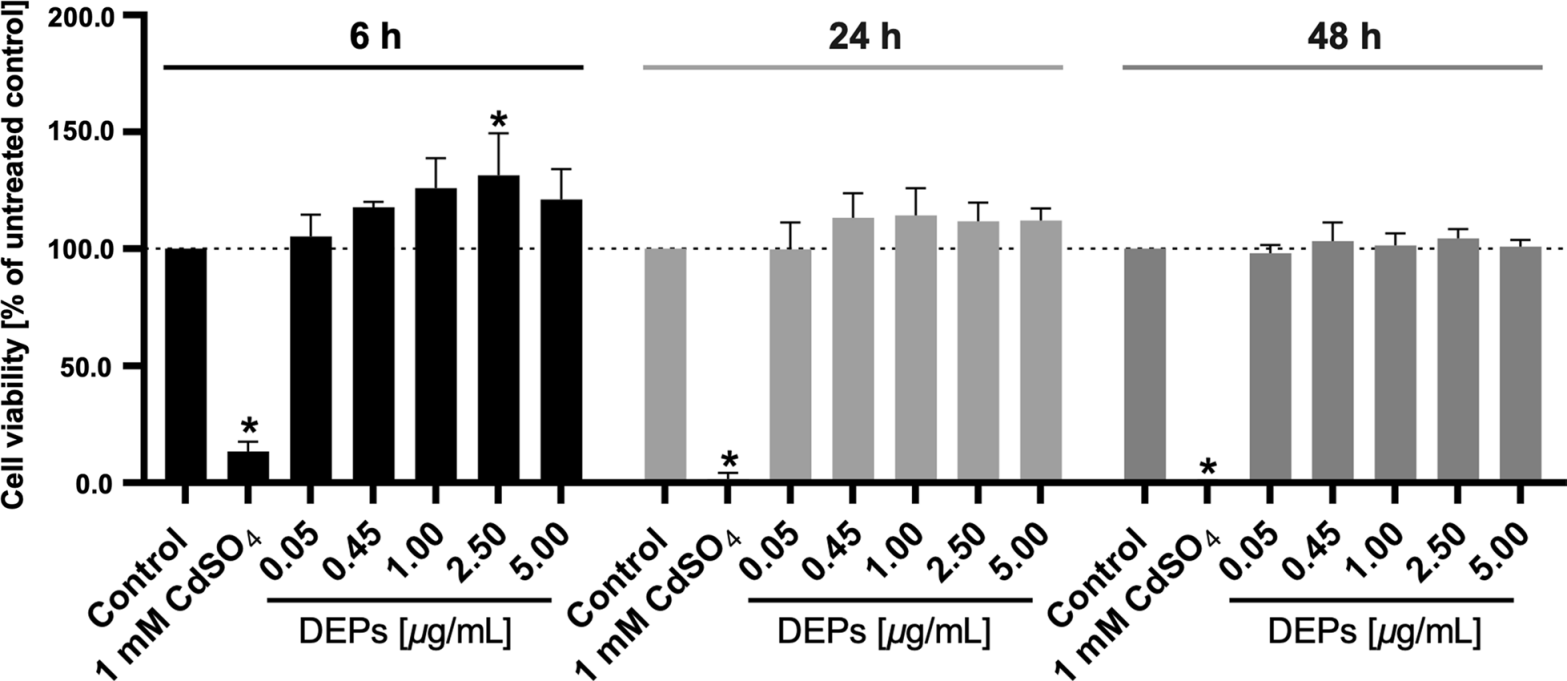
Effect of DEPs on BeWo trophoblast viability. Cell viability is assessed after treatment with 0.05–5 μg/mL DEPs for 6, 24, and 48 h using the MTS assay. 1 mM CDSO_4_ is applied as a positive control for cytotoxicity. The dotted line indicates 100% viable cells. Data represent the mean percentages of viable cells compared to the untreated control (SD) of three independent biological experiments with three technical repeats each. One-way ANOVA with Tukey’s multiple comparison correction is performed to find significance between the untreated control group and different treatment groups, *p < 0.05 is considered significant. *ANOVA* analysis of variance, *DEP* diesel exhaust particle, *SD* standard deviation

To verify the absence of cytotoxicity and possible damage to the placental barrier by DEP exposure, an MTS viability assay was performed on BeWo trophoblast cells, representing the syncytiotrophoblast layer of the placental barrier. DEP concentrations from 0 to 5 µg/mL were applied to the cells for up to 48 h (a multiple of the chosen ex vivo conditions of 0.45 µg/mL DEPs for 6 h). Overall, the cell viability was not affected. Only the exposure to 2.5 µg/mL of DEPs for 6 h led to a significant increase in BeWo viability (Fig. 2).

### Ex vivo placental uptake and translocation of carbon particles

**Fig. 3:**
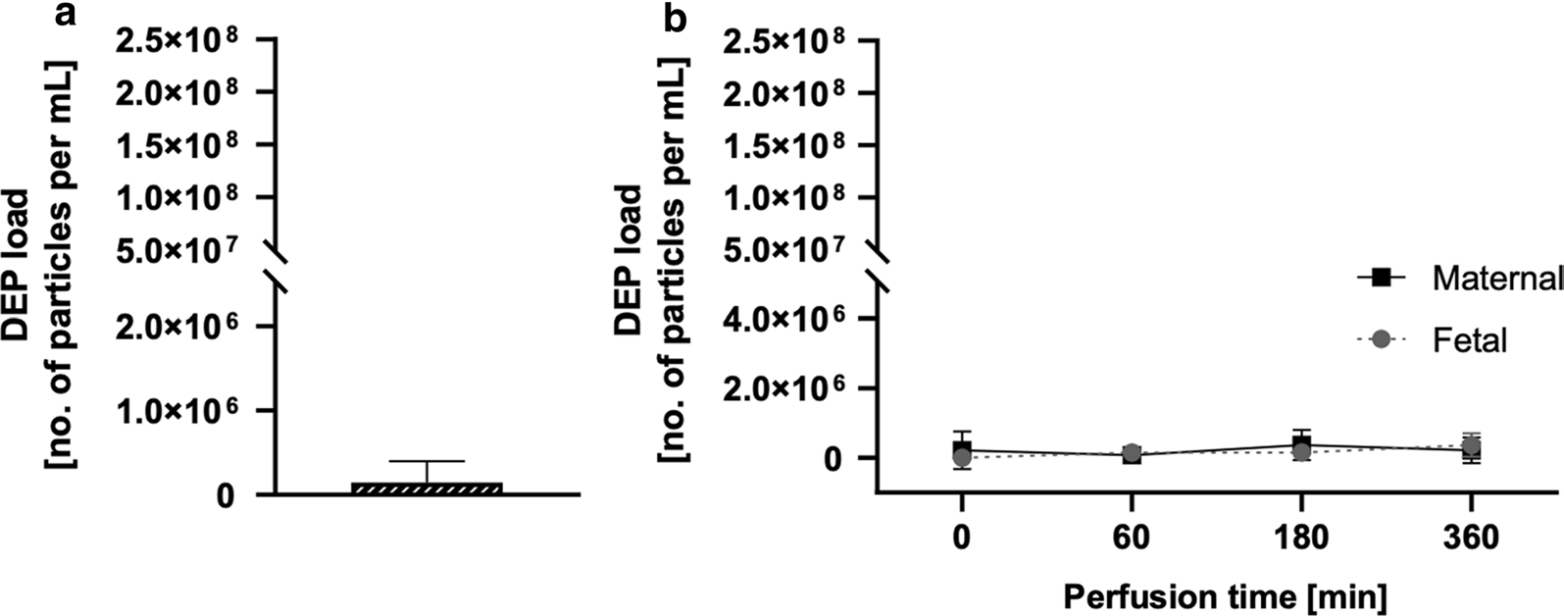
Determination of carbon particle background during placental perfusion with plain perfusion medium. Placental perfusion is performed for 6 h with empty PM, and the carbon particle content is determined using femtosecond pulsed laser illumination in PM before perfusion (**a**) as well as in the maternal and fetal perfusates over time (**b**). Data represent the mean (SD) of three technical replicates (**a**) and two independently perfused placentae (**b**). p < 0.05 is considered statistically significant (* denotes differences in carbon particle concentration determined in fetal perfusates over time compared to the initial amount) as analyzed by one-way ANOVA with Tukey’s multiple comparison correction. *ANOVA* analysis of variance, *PM* perfusion medium, *SD* standard deviation

We used our previously established imaging technique based on the non-incandescence-related white light (WL) generation by carbonaceous particles under near-infrared femtosecond pulsed illumination [28] to study the presence and location of carbon particles in human perfused placentae and obtained maternal and fetal perfusates. First, control perfusions were performed for up to 6 h with plain perfusion medium (PM) to determine a potential carbon particle background from external contamination (Fig. 3). We only detected low particle concentrations (SD) in the empty PM (1.5 × 10^5^ (2.5 × 10^5^) particles/mL) before perfusion (Fig. 3a), and fetal and maternal perfusion values did not significantly increase during the 6 h of perfusion with empty PM (Table 2). In addition, no significant difference in placental particle load was observed following 6 h of perfusion with plain PM (Additional file 1: Table S1).

**Table 2:** Perfusion kinetics of carbon particles in fetal and maternal perfusates determined by femtosecond pulsed laser illumination

Carbon particle load (SD)	Perfusion time ( min)	Fetal perfusates (no. particles/mL)	*p*-value^a^	Maternal perfusates (no. particles/mL)	*p*-value^b^
Exposed (N = 4)	0	7.7 × 10^5^ (8.8 × 10^5^)	/	1.7 × 10^8^ (3.6 × 10^7^)	/
	60	12 × 10^5^ (8.7 × 10^5^)	0.7258	1.1 × 10^8^ (2.5 × 10^7^)	< 0.0001*
	180	8.1 × 10^5^ (13 × 10^5^)	0.9997	1.1 × 10^8^ (2.4 × 10^7^)	< 0.0001*
	360	3.7 × 10^5^ (6.2 × 10^5^)	0.7258	1.0 × 10^8^ (1.7 × 10^7^)	< 0.0001*
Control (N = 2)	0	0.0	/	2.2 × 10^5^ (5.4 × 10^5^)	/
	60	1.5 × 10^5^ (2.3 × 10^5^)	0.6930	0.7 × 10^5^ (1.8 × 10^5^)	0.9203
	180	1.5 × 10^5^ (2.3 × 10^5^)	0.6930	3.7 × 10^5^ (4.3 × 10^5^)	0.9203
	360	3.7 × 10^5^ (3.3 × 10^5^)	0.0550	2.2 × 10^5^ (3.7 × 10^5^)	> 0.9999

*ANOVA* analysis of variance, *SD* standard deviation

^*^p < 0.05 was considered statistically significant as analyzed by one-way ANOVA with Tukey’s multiple comparison correction

^a^Differences in carbon particle concentration determined in fetal perfusates over time compared to the initial amount

^b^Differences in carbon particle concentration determined in maternal perfusates over time compared to the initial amount. Data represent the mean DEP load (SD) in fetal and maternal perfusates collected after different perfusion times of 4 (exposed), and 2 (control) independently perfused placentae

**Fig. 4:**
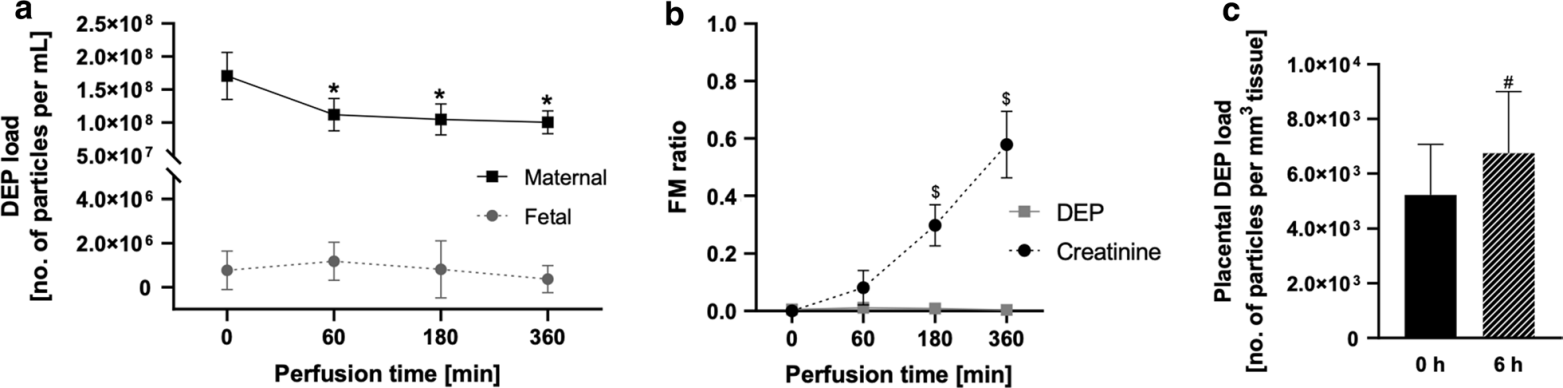
Human placental perfusion with 0.45 µg/mL DEPs for 6 h. The carbon particle content is determined with femtosecond pulsed laser illumination in maternal and fetal perfusates over time (**a**). Next, FM ratios are calculated for each time point and compared to FM ratios of the reference compound creatinine (**b**). Additionally, the carbon particle content is measured in tissue samples of each placenta collected before and after perfusion (**c**). Data represent the mean (SD) of four independently perfused placentae with medium containing 0.45 µg/mL DEP. *p* < 0.05 is considered statistically significant (* denotes differences in carbon particle concentration determined in maternal perfusates over time compared to initial amount; ^$^ denotes differences in FM ratio between DEP and creatinine, ^#^ denotes differences in placental carbon particle load before and after DEP perfusion) as analyzed by one-way ANOVA with Tukey’s multiple comparison correction and paired *t*-test. *ANOVA* analysis of variance, *DEP* diesel exhaust particle, *FM* fetal–maternal, *SD* standard deviation

Carbon particle uptake, accumulation, and translocation were assessed in the dynamic ex vivo placental perfusion model for up to 6 h (Fig. 4, Table 2). To ascertain barrier integrity and sufficient overlap between the maternal and fetal circulation, creatinine was added as a reference compound for passive diffusion in all perfusion experiments (Additional file 1: Figure S2). Creatinine showed the expected pharmacokinetic profile with a slow placental transfer that approaches but does not completely reach equilibrium after 6 h of perfusion [33]. Moreover, the addition of DEPs did not influence creatinine transfer. In the perfusion studies with DEPs (0.45 µg/mL), the maternal carbon particle concentration (SD) significantly decreased from 1.7 × 10^8^ (3.6 × 10^7^) particles per mL perfusate at start to 1.0 × 10^8^ (1.7 × 10^7^) particles per mL perfusate after 6 h of perfusion. On the other hand, the mean carbon particle concentration in the fetal perfusates did not change significantly over time (Fig. 4a, Table 2).

In contrast to the passive transfer of creatinine, fetal–maternal (FM) ratios for DEPs remained close to zero during 6 h of perfusion (Fig. 4b). Quantification of carbon particles in placental tissue samples before (collected from adjacent non-perfused cotyledon) and after perfusion (collected from perfused cotyledon) revealed an accumulation of carbon particles with a significant increase in placental particle load (SD) from 5.2 × 10^3^ (1.8 × 10^3^) particles per mm^3^ placental tissue before perfusion to 6.8 × 10^3^ (2.2 × 10^3^) particles per mm^3^ placental tissue after perfusion (Fig. 4c, Additional file 1: Table S1). Adsorption to the perfusion device was ruled out by studying the perfusion of DEPs in the absence of placental tissue, which demonstrated that DEPs stayed in suspension over 6 h of perfusion without a significant decrease in the particle count (Additional file 1: Figure S3).

### Cellular distribution and colocalization imaging

**Fig. 5:**
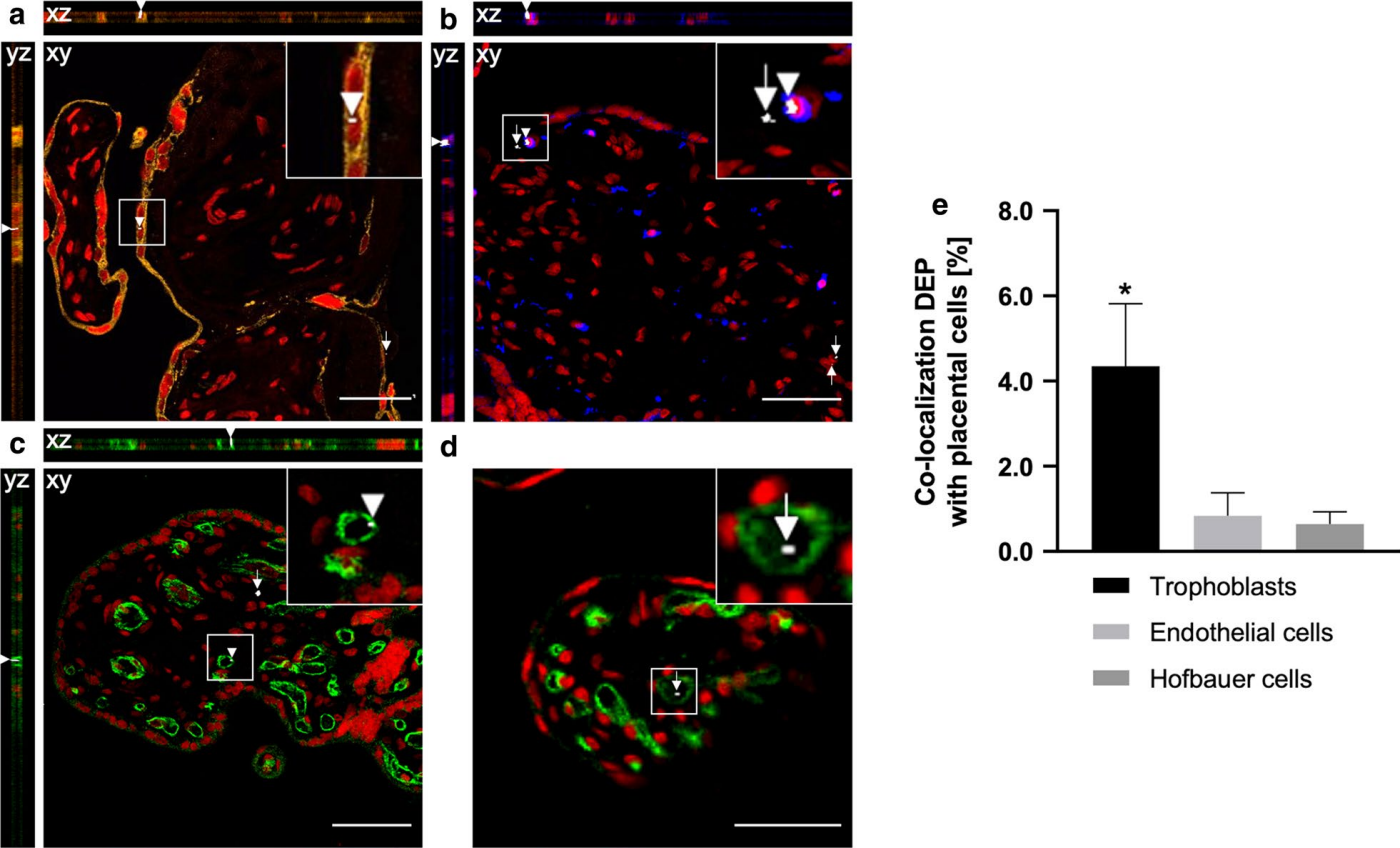
Uptake and localization of carbon particles in placental villous tissue. Orthogonal views and images of placental tissue sections after 6 h of perfusion with 0.45 μg/mL DEPs (**a**–**d**). Trophoblast cells are stained with anti-cytokeratin (AE1/AE3, orange) (**a**), placental macrophages with anti-CD68 (blue) (**b**) and endothelial cells with anti-CD31 (green) (**c**, **d**). Syto 61 Red (red) was used as a nuclear counterstain (**a**–**d**). Few carbon particles are also detected inside fetal microvessels (**d**). The carbon particles are imaged under femtosecond pulsed illumination (white; arrowheads and arrows indicate carbon particles colocalized or not with the stained cell type, respectively). Approximately 22 images with a 512 × 512-pixel resolution were acquired throughout the stained placental section with an optical slice thickness of 1 µm using a pixel dwell time of 4.10 µs. xz and yz represent cross-sectional views of the optical volume corresponding to the image stack collected from a colocalized particle. Presented images are representative of all investigated samples. Scale bars: 30 µm (**a**), 50 µm (**b**, **c**), and 20 µm (**d**). The number of detected particles colocalized with a specific cell type was quantified using the Manders’ coefficient (**e**). Data represent the colocalization coefficient (SD) of four independently perfused placentae with medium containing 0.45 µg/mL DEPs (225 images/placenta/cell type). One-way ANOVA with Tukey’s multiple comparison correction is performed to find significance in the percentage colocalization between the different cell types, *p < 0.05 is considered significant. *ANOVA* analysis of variance, *DEP* diesel exhaust particle, *SD* standard deviation

The cellular distribution of carbon particles in the placental villous tissue was evaluated by colocalization of carbon particles with the following placental cell types; trophoblast cells, endothelial cells, and placental macrophages (Hofbauer cells) (Fig. 5a–c). Carbon particles were visualized by probing their WL generation as reported previously [28], and internalization was observed for all the investigated placental cell types after perfusion with empty PM (Additional file 1: Figure S4) and DEPs (Fig. 5a–c). The orthogonal projections confirm the placental embedment of the particles and, with this, preclude external contamination. We were also able to detect some carbon particles inside fetal capillaries (Fig. 5d), confirming that the complete transfer of carbon particles across the human placenta is feasible. In addition, the number of detected carbon particles that colocalize with a specific placental cell type was further quantified by determining the Manders’ colocalization coefficient by using the JACoP ImageJ plug-in [34, 35]. The carbon particles showed an overlap (SD) with (i) trophoblasts of 4.4 (1.5)%, (ii) endothelial cells of 0.8 (0.5)% and (iii) macrophages of 0.6 (0.3)% (Fig. 5e) in the DEP perfused placental tissue. A similar distribution was observed for the control samples after perfusion with empty medium. The carbon particles from ambient exposure showed an overlap (SD) with (i) trophoblasts of 5.6 (1.9)%, (ii) endothelial cells of 1.1 (0.9)% and (iii) macrophages of 0.3 (0.2)%.

## Discussion

Seeing the ability of ambient air pollution particles to reach the maternal systemic circulation upon inhalation exposure during pregnancy, we investigated the translocation and biodistribution of DEPs as a model particle for combustion-derived pollution in human perfused placental tissue. To this end, we employed the current gold standard ex vivo placental perfusion model [27] to assess placental transfer of DEPs towards the fetal circulation in an intact human tissue under physiologically-relevant perfused conditions. Placental perfusion studies were performed for up to 6 h, which has been previously shown to be sufficient to detect placental translocation of various nanoparticles [36–40].

To achieve meaningful results and avoid potential adverse effects due to overload conditions, we aimed to investigate realistic exposure concentrations. In the current study, we employed a DEP concentration extrapolated from the carbon particle load found in placental tissue of high exposed mothers to an average BC concentration ranging between 1.70 to 2.42 μg/m^3^ during pregnancy [14]. In BeWo trophoblast cells, this concentration did not evoke any cytotoxic effects for exposure times up to 48 h, indicating that the DEPs do not adversely affect the trophoblast layer of the placental barrier. Moreover, the absence of maternal–fetal leakage of PM further corroborates that the particles did not compromise barrier integrity during the 6 h of perfusion experiments. The detected significant increase in metabolic activity of BeWo cells (2.5 μg/mL for 6 h) could be explained by hormesis. A biphasic cell response where lower exposure levels induce cell metabolism, while higher or more extended exposure scenarios can harm the cells [41, 42]. However, it remains to be determined if higher and/or prolonged DEP exposure could adversely affect trophoblast viability.

In general, passive diffusion mechanisms did not appear to be affected by DEP exposure, as evidenced by the similar translocation profiles of creatinine in the presence or absence of particles. These observations collectively suggest that DEPs observed in the fetal microvessels or fetal circulation will have crossed an intact placental barrier. One drawback of our study is that we cannot distinguish between the DEPs added to the ex vivo perfusion experiment and the ambient combustion-related particulates that already accumulated in placental tissue during real-life gestational exposure and which may potentially be released from the tissue upon perfusion. Yet, fluorophore-labeling of the added carbon particles for differentiation might cause wrong conclusions due to effects of the label on biointeractions [43] or loss of the label [44]. However, only low particle concentrations were detected in control perfusions with plain PM performed for up to 6 h, indicating that ambient particulates already present in the placental tissue or PM should not profoundly confound the experimental measurements. No information on maternal residential BC exposure was available, yet the average carbon particle count in the placental biopsies collected before (i.e., 5.2 × 10^3^ particles per mm^3^ tissue) and after (i.e., 6.8 × 10^3^ particles per mm^3^ tissue) perfusion were not that different from the placental BC load of mothers exposed to relatively low ambient BC concentrations (average whole pregnancy concentrations ranging between 0.63 and 0.96 µg/m^3^ in the Belgian study area) (i.e., 9.5 × 10^3^ particles per mm^3^ tissue) [14]. Moreover, the colocalization experiments in empty control and DEP perfused placental tissue show comparable colocalization coefficients indicating a similar placental distribution of environmental carbon particles (from ambient exposure only) and added carbon particles (ex vivo DEP perfusion). This further supports that the estimated applied DEP dose is in the range of a real-life exposure condition.

Here, we used our previously established imaging technique based on the non-incandescence-related white light (WL) generation by carbonaceous particles under near-infrared femtosecond pulsed illumination [28] to study the presence and location of DEPs in human perfused placentae and maternal and fetal perfusates. This biocompatible and label-free detection technique allows (i) the direct analysis and visualization of carbonaceous particles in biological matrices without the need for sample preparation (e.g., macrophage isolation) and labeling, (ii) the preservation of the biological context enabling discrimination between cellular structures, (iii) the simultaneous detection and colocalization of various labeled cellular compartments and carbonaceous particles and (iv) the detection of carbonaceous particles with high sensitivity and specificity. Under femtosecond pulsed illumination, the carbon particles generate a strong, saturated WL emission compared to the fluorescence signal originating from the fluorophore-labeled placental cell types [28]. Accordingly, the Manders’ colocalization coefficient was employed to quantify the colocalization of carbon particles with the placental cell types of interest since this metric is independent of signal proportionality [35, 45].

After 6 h of perfusion, we identified a significant uptake of carbon particles into human placental tissue ex vivo, which is in line with previous observations in vivo [14, 15, 23]. In general, particulates are effectively ingested by macrophages as the first line of defense to clear them from the system [46]. In this regard, the presence of inhaled ambient combustion-related particulates has been reported in airway macrophages [47] and macrophage-enriched placental cells [15]. Likewise, we observed the colocalization of carbon particles with placental macrophages. Yet, our colocalization analysis shows the preferential uptake of carbon particles by trophoblasts, which form the key cellular layer of the placental barrier, the so-called syncytiotrophoblast, as previously also described for other nanosized particles [37, 48, 49]. This is likely due to (i) the direct contact between this epithelial barrier layer and the maternal blood and (ii) the layer’s syncytial nature (fusion of multiple cells without cell boundaries), which largely restricts paracellular transport. Accumulation of carbon particles in the syncytiotrophoblast may interfere with its many essential functions for maternal–fetal health, and therefore deserves increasing attention (as reviewed in [13]).

The colocalization of carbon particles with endothelial cells demonstrates that some particles can overcome the syncytiotrophoblast layer and potentially cross the endothelial layer as well, which is generally less restrictive than the syncytiotrophoblast. Indeed, few particles were detected inside fetal microvessels. But carbon particle concentrations did not significantly increase in the fetal circulation after 6 h of perfusion, despite (i) a three orders of magnitude higher particle concentration in the maternal perfusate and (ii) the thinnest state of the actual cellular barrier at term pregnancy [50, 51]. Therefore, our study suggests that the transfer of carbon particles across the human placenta is possible yet highly limited (as compared to other ex vivo particle translocation studies with particles in similar size ranges but different material [52]). However, only short exposure times (6 h) (due to tissue degradation [53]) were studied, and low deposition of particles in the perfusion system as well as variations between individual measurements are possibly masking low particle counts. In addition, only a limited amount of perfusions (two control perfusions and four DEPs perfusions) was performed due to the high complexity and low success rate of this method [52], nevertheless, the obtained results were highly consistent even from this low number of independent experiments. Further verification of realistic direct chronic exposure of the fetus to combustion-derived particles could be pursued from human cord blood samples to corroborate the maternal–fetal transfer of carbon particles further.

## Conclusions

While our findings support fetal transfer and thus potential direct fetotoxicity of DEPs, the considerable accumulation of particles in the syncytiotrophoblast layer highlights the need for more intensified research into the mechanisms of the indirect developmental toxicity of ambient particulate matter mediated by possible adverse effects of these particles on placental tissue function and signaling pathways. Besides potential interference with essential functions of trophoblast cells (e.g., endocrine, metabolic, transport, or barrier functions), uptake of carbon particles in placental macrophages could interfere with important inflammatory and/or immune functions, in particular upon repeated prolonged exposures. Understanding placental translocation and toxicity of combustion-related particulates can help gain more insight into the observed detrimental effects of ambient particulate air pollution on fetal development and reduce adverse pregnancy outcomes and the risks of damaging in utero exposure and disease development later in life.

## Methods

### DEPs dispersion and characterization

The DEPs were obtained from the National Institute for Standards and Technology (NIST^®^, SRM^®^1650b, https://www-s.nist.gov/srmors/certificates/1650b.pdf). Stock dispersions of 45 µg/mL were prepared in PM (see below for composition). The stock suspension was dispersed using a probe sonicator operating at 230 V/50 Hz (Branson Sonifier 250, Branson Ultrasonic Co., probe diameter of 6.5 mm, the maximum peak-to-peak amplitude of 247 μm). The DEPs stock suspension was sonicated for 20 min on ice at 5% of the maximum peak-to-peak amplitude, corresponding to a specific acoustic energy of 7 W, as determined by a calorimetric method for probe sonicator calibration. Before each characterization and perfusion experiment, the stock suspension was sonicated in a water bath (10 min, Sonorex RK156, Bandelin) and vortexed for 1 min. For perfusion experiments, the stock suspension was immediately diluted in PM under stirring conditions. Dilutions for characterization and other experiments were prepared by adding respective amounts from stock suspension dropwise into a falcon containing PM while vortexing. At working concentration, suspensions were again bath sonicated for 5 min at RT and vortexed for 1 min immediately before characterization. DEP suspensions were characterized by dynamic light scattering (DLS) (hydrodynamic size and zeta potential) using a Zetasizer (Nano ZS90, Malvern Instruments) equipped with a 4.0 mW He–Ne laser operating at 632.8 nm and with an avalanche photodiode detector. A set of five measurements was conducted with a 4.5 µg/mL DEPs suspension at 25 °C, with an automatic setting of acquisitions for each set.

### Limulus amebocyte lysate (LAL) assay

The Pierce LAL Chromogenic Endotoxin Quantitation Kit (Thermo Scientific, 88282) was performed according to the manufacturer’s guidelines with an assay sensitivity of 1–0.1 EU/mL. In brief, the DEPs samples, at 0.45, 4.5, and 45 μg/mL, were mixed with the LAL reagent supplied in the test kit and incubated at 37 °C for 10 min. A chromogenic substrate solution was then mixed with the LAL-sample mixture and incubated at 37 °C for an additional 6 min. The reaction was then stopped with a stop reagent provided with the kit, and absorbance was measured at 405–410 nm on a plate reader (Mithras^2^ LB 943, Berthold Technologies GmbH, Zug, Switzerland).

### Cell culture

The human placental choriocarcinoma cell line BeWo b30 was kindly provided by Prof. Dr. Ursula Graf-Hausner (Zurich University of Applied Science) with permission from Dr. Alan L. Schwartz (Washington University School of Medicine, MO, USA). BeWo cells were routinely cultivated in Ham’s F-12K medium supplemented with 10% fetal calf serum (FCS, Invitrogen, Basel, Switzerland), 1% pen/strep (Gibco, Luzern, Switzerland), and 2 mM l-Glutamine (Gibco, Luzern, Switzerland). BeWo cells were sub-cultured twice a week using a trypsin–EDTA solution and cultivated in a humidified incubator at 37 °C with 5% CO_2_ atmosphere.

### MTS viability assay

The cytotoxicity of the DEPs was tested in vitro using the MTS viability assay. BeWo cells were seeded in a 96-well plate (10,000 cells per well) for 24 h and treated with different concentrations of DEPs ranging from 0 to 5 μg/mL. Cells without treatment were used as a negative control, and as a positive control, 1 mM CdSO_4_ was applied. After 6, 24, or 48 h of incubation at 37 °C and 5% CO_2_, an MTS assay (CellTiter96^®^ AQueous One Solution Cell Proliferation Assay, Promega, Switzerland) was performed according to the manufacturer’s instructions. Results were presented as the mean percentage of the untreated control from three independent experiments.

### Ex vivo placental perfusion

Placentae were obtained from uncomplicated pregnancies after cesarean section from the Kantonsspital St. Gallen and Hirslanden Klinik Stephanshorn St. Gallen with written informed consent from the expecting mothers. The study was approved by the local ethics committee (EKOS 10/078; PB-2018-00069) and performed according to the principles of the Declaration of Helsinki. The recirculating dually perfused ex vivo placental perfusion model was performed as previously described [37, 54]. The PM was DMEM culture medium (D1145), which was diluted with Earl's buffer (E3024) in equal parts (1:2) and further supplemented with bovine serum albumin (BSA; 10 g/L), dextran 40 (10 g/L), sodium heparin (17.5 mg/L ≈ 3150 USP units/L) and amoxicillin (250 mg/L) (medium and all supplements were obtained from Sigma, Buchs, Switzerland). Criteria for successful perfusion were: (i) the pre-perfusion of the tissue showed no leakage, (ii) the leakage (fetal to maternal) was less than 4 mL/h during the translocation experiment, and (iii) the pH remained constant during the experiment (7.2–7.4). Moreover, the passive diffusion reference marker creatinine was added to all perfusions (final concentration of 100 mg/L in the maternal reservoir) to demonstrate sufficient overlap between maternal and fetal circulation. Perfusions were performed only with perfusion medium (N = 2) and with perfusion medium containing DEPs (N = 4; only added to maternal circulation; final concentration 0.45 µg/mL). Samples (5 mL) from maternal and fetal perfusates were collected at 0, 1, 3, and 6 h of perfusion and analyzed immediately for pH, creatinine, glucose, and lactate using a blood gas analyzer (epoc^®^ Blood Analysis system with BGEM test cards; Epocal Inc., Ottawa, Canada). Afterward, supernatants were stored at − 20 °C for further analysis. Placental tissue samples were collected before (from adjacent non-perfused cotyledon) and after perfusion (from perfused cotyledon) and stored at RT in 4% paraformaldehyde for further analysis.

### Detection of carbon particles in perfusates and tissue

Carbon particles present in the ex vivo perfused placental tissue and perfusates were detected using a specific and sensitive detection technique based on the non-incandescence-related WL generation of the particles under femtosecond pulsed illumination as previously described [14, 28, 55]. Placental biopsies were collected before (from adjacent non-perfused cotyledon) and after each perfusion (from perfused cotyledon) experiment, fixed in 4% formaldehyde for a minimum of 24 h and embedded in paraffin. 4 µm sections were cut using a microtome (Leica Microsystems, UK), floated onto charged glass slides (Super-Frost Plus, Fisher Scientific, USA) and dried overnight at 37 °C. Carbon particles present in the placental tissue and perfusates were detected using our specific and sensitive detection technique based on the non-incandescence-related white light (WL) generation of the particles under femtosecond pulsed illumination [28]. Images of the placental sections and z-stacks of the perfusates were collected at RT using a Zeiss LSM 880 (Carl Zeiss, Germany) equipped with a femtosecond pulsed laser (810 nm, 150 fs, 80 MHz, MaiTai DeepSee, Spectra-Physics, USA) tuned to a central wavelength of 810 nm using an EC Plan-Neofluar 10×/0.30 and an LD C-Apochromat 40×/1.1 W Korr UV–Vis-IR objective (Carl Zeiss, Germany). Two-photon induced WL emission of carbon particles was acquired in the non-descanned mode after spectral separation and emission filtering using 400–410 nm and 450–650 nm band-pass filters. The resulting tile scans of placental tissue were recorded with a 1.66 μm pixel size and 4.10 μs pixel dwell time. A total of 15 placental sections was imaged per sample. Each perfusate sample was aliquoted at 200 μL/well in an Ibidi *μ*-slide eight well, and z-stacks were collected from 50 till 100 μm above the bottom of the well plate. The resulting z-stacks have an imaging volume of 212.55 × 212.55 × 50.29 μm^3^, and a pixel dwell time of 2.05 μs. In total, 345 images per perfusate sample were obtained by recording z-stacks with a 0.44 μm slice thickness at three different locations in the aliquot. The images were acquired by ZEN Black 2.0 software (Carl Zeiss, Germany).

The number of carbon particles in the obtained tile scans and z-stacks was determined by using a peak-find algorithm, which counts connected pixels above a certain threshold value. Here, threshold values of 0.5% and 40% lower than the highest pixel intensity value of the narrow second harmonic generation channel (405/10) and two-photon excited autofluorescence channel (550/200), respectively, were chosen. The average amount of particles detected in the placental tissue tile scans and perfusate z-stacks was normalized to the placental tissue area and the imaging volume, respectively. Finally, the results were expressed as the number of detected carbon particles per mm^3^ placental tissue and mL perfusate.

### Cellular carbon particle distribution and colocalization imaging

Cellular distribution of carbon particles in perfused (0.45 µg/mL) human placentae was evaluated by staining trophoblasts, endothelial cells, and Hofbauer cells in triplicate. For marker expression analysis, 4 µm thick placental paraffin sections of perfused placental tissue were deparaffinized in xylene and rehydrated in a graded ethanol series to be submerged in PBS. Next, the sections were placed in a citrate buffer solution (10 mM, pH 6.0) for 40 min at 97 °C for antigen retrieval. Endogenous peroxidase activity was quenched by immersing the sections in 0.3% H_2_O_2_ in PBS for 10 min. After blocking non-specific binding sites with protein block (X0909, Agilent Dako, USA) for 60 min, tissue sections were probed with mouse monoclonal antibody against (i) human cytokeratin EA1/EA3 (1:50, N1590, Agilent, USA), (ii) human CD31 (1:100, M0823, Agilent, USA) or (iii) human CD68 (1:100, M0814, Agilent, USA) overnight at 4 °C. After washing, the tissue sections were incubated with an Alexa Fluor^®^ 555 conjugated goat-anti mouse secondary antibody (1:500, A2122, Invitrogen, USA). All antibodies were diluted in 10% protein block/PBS. SYTO™ 61 Red (1:1000, S11343, Invitrogen, USA) was used as a nuclear counterstain.

Tile scans of 5 × 5 images of stained tissue sections were acquired using the microscope set-up as described above now using a Plan-Apochromat 20×/0.8 (Carl Zeiss, Germany) objective, resulting in a field of view of 2125.48 × 2125.48 µm^2^ with a 2560 × 2560 pixel resolution (0.83 × 0.83 µm^2^ pixel size) and a pixel dwell time of 4.10 µs. Alexa Fluor^®^ 555-labeled placental cells (i.e., trophoblasts, endothelial cells, macrophages) and SYTO™ 61 Red-labeled nuclei were excited by using a 0.64 mW 543 He–Ne laser and a 5 mW 633 He–Ne laser, respectively. Band-pass filters 490–600 nm and 650–750 nm were used for filtering the emission signal from the labeled placental cells and nuclei, respectively. As described above, carbon particles were excited and visualized using a Plan-Apochromat 20×/0.8 (Carl Zeiss, Germany) objective. The images were acquired by ZEN Black 2.0 software (Carl Zeiss, Germany). To quantify the colocalization of carbon particles with the markers of interest, nine overview images per marker were collected in three stained sections from each DEP-perfused placenta (*N* = 4). Manders’ overlap coefficients, describing the percentage of particles associated with the labeled cells, were calculated using the Fiji Just Another Colocalization Plugin (JACoP) in Fiji (Image J v2.0, Open source software, http://fiji.sc/Fiji) [50]. Prior to analysis, a threshold was set to the estimated background value. The colocalization coefficient was defined as the percentage of carbon particles overlapping with the labeled placental cells. Obtained coefficients are not dependent on the relative intensities of each channel and cross-talk between the channels was found to be negligible. In addition, optical sectioning in the z-direction throughout the placental tissue was performed to (i) show the colocalization of carbon particles with the placental cells of interest and (ii) to confirm placental embedment and with this preclude external contamination. Approximately 22 images with a 512 × 512-pixel resolution were acquired throughout the stained placental section with an optical slice thickness of 1 µm using a pixel dwell time of 4.10 µs. Orthogonal xz- and yz-projections were made using Fiji. Channel registration was corrected using TetraSpeck™ Fluorescent Microspheres (T7279, Invitrogen, USA), and the channels were manually aligned to overlap.

### Data processing and statistics

Data is represented as mean (SD) of at least three independent experiments (unless stated otherwise) and analyzed using the commercially available GraphPad Prism software (GraphPad Prism 8, GraphPad Software Inc., USA). A paired *t*-test or analysis of variance (ANOVA) with Tukey’s post-test for multiple comparisons was performed. Differences were considered statistically significant at *p* < 0.05.

## Supplementary Information

**Additional file 1: Table S1.** Placental carbon particle load determined by femtosecond pulsed laser illumination. **Fig S1.** Size distribution by intensity of DEPs (0.45 μg/mL) in PM. **Fig S2.** Perfusion profiles and FM ratio of the reference compound creatinine. **Fig S3**. DEP absorbance to the perfusion system components. **Fig S4.** Localization of carbon particles in placental villous tissue.

## Data Availability

The datasets used and/or analyzed during the current study are available from the corresponding author on reasonable request.

## Abbreviations

ANOVA: Analysis of variance
BC: Black carbon
BET: Brunauer–Emmet–Teller method
BeWo b30: Human placental choriocarcinoma cell line
BGEM: Blood gas electrolyte and metabolite
BSA: Bovine serum albumin
CdSO_4_: Cadmium sulfate
CO_2_: Carbon dioxide
d(0.5): Mean particle-size indicating the diameter below which 50% of the volume is present
d(0.9): Mean particle-size indicating the diameter below which 90% of the volume is present
ddH_2_O: Double distilled water
DEP: Diesel exhaust particle
DLS: Dynamic light microscopy
DMEM: Dulbecco’s modified eagle medium
EC: Enhanced contrast
EDTA: Ethylenediaminetetraacetic acid
EU/mL: Endotoxin units per milliliter
FCS: Fetal calf serum
FM: Fetal–maternal
g: Gram
h: Hours
H_2_O_2_: Hydrogen peroxide
hCG: Human chorionic gonadotropin
He–Ne: Helium–neon
Hz: Hertz
JACoP: Just Another Colocalization Plugin
L: Liter
LAL: Limulus amebocyte lysate
LD: Long working distance
LSM: Laser scanning microscope
m^2^: Square meter
m^3^: Cubic meter
mg: Milligram
min: Minute
mL: Milliliter
mm: Millimeter
mm^3^: Cubic millimeter
mM: Millimolar
mV: Millivolt
µg: Microgram
µm: Micrometer
µm^3^: Cubic micrometer
µs: Microsecond
nm: Nanometer
PBS: Phosphate buffered saline
PDI: Polydispersity index
PM: Perfusion medium
PM_2.5_: Particulate matter smaller than 2.5 µm in diameter
RT: Room temperature
SD: Standard deviation
USP: United States Pharmacopeia
UV–Vis-IR: Ultraviolet–visible-infrared
V: Volt
W: Watt
WL: White light
