# Supplementary material for: Label-free detection of uptake, accumulation, and translocation of diesel exhaust particles in ex vivo perfused human placenta
Source: J Nanobiotechnology. 2021 May 17;19:144. doi: 10.1186/s12951-021-00886-5 (PMC8130319; doi:10.1186/s12951-021-00886-5)
Supplement: Supplementary file 1 — Additional file 1: Table S1. Placental carbon particle load determined by femtosecond pulsed laser illumination. Fig S1. Size distribution by intensity of DEPs (0.45 μg/mL) in PM. Fig S2. Perfusion profiles and FM ratio of the reference compound creatinine. Fig S3. DEP absorbance to the perfusion system components. Fig S4. Localization of carbon particles in placental villous tissue. [file 12951_2021_886_MOESM1_ESM.docx]

Additional Information

**Label-Free Detection of Uptake, Accumulation, and Translocation of Diesel Exhaust Particles in Ex Vivo Perfused Human Placenta**

*Eva Bongaerts^1,§^, Leonie Aengenheister^2,§^, Battuja B. Dugershaw^2^, Pius Manser^2^, Maarten B.J. Roeffaers^3^, Marcel Ameloot^4^, Tim S. Nawrot^1,5^, Hannelore Bové^1,4^*, Tina Buerki-Thurnherr^2,^**

1. Centre for Environmental Sciences, Hasselt University, Agoralaan Building D, 3590 Diepenbeek, Belgium.
2. Laboratory for Particles-Biology Interactions, Empa, Swiss Federal Laboratories for Materials Science and Technology, St. Gallen, Switzerland.
3. Centre for Surface Chemistry and Catalysis, KU Leuven, Leuven, Belgium
4. Biomedical Research Institute, Hasselt University, Agoralaan Building C, 3590 Diepenbeek, Belgium
5. Department of Public Health and Primary Care, KU Leuven, Herestraat 49 - box 703, 3000 Leuven, Belgium.

^§^ These authors contributed equally.

* Address correspondence:

Tina Buerki-Thurnherr, PhD, Laboratory for Particles-Biology Interactions, Empa, Swiss Federal Laboratories for Materials Science and Technology, St. Gallen, Switzerland. Phone: +41 79 6454145. Email: tina.buerki@empa.ch.

Hannelore Bové, PhD, Centre for Environmental Sciences, Hasselt University, Agoralaan Building D, 3590 Diepenbeek, Belgium. Phone: +32 11 268381. Email: hannelore.bove@uhasselt.be.

**Experimental Section**

**Table S1.** Placental carbon particle load determined by femtosecond pulsed laser illumination

| Carbon particle  load (SD) | Perfusion time  [min] | Placental carbon particle load  [no. particles per mm^3^ tissue] | *p*-value |
| --- | --- | --- | --- |
| **Exposed (N=4)** |  |  |  |
|  | 0 | 5.2 x 10^3^ (1.8 x 10^3^) | / |
|  | 360 | 6.8 x 10^3^ (2.2 x 10^3^) | 0.0487* |
| **Control (N=2)** |  |  |  |
|  | 0 | 3.2 x 10^3^ (3.4 x 10^2^) | / |
|  | 360 | 4.1 x 10^3^ (1.9 x 10^2^) | 0.4894 |

The placental particle concentration was determined in tissue samples of each placenta collected before and after perfusion with 0.45 µg/mL DEPs for 6 h. Data represent the mean carbon particle load (SD) in placental tissue samples collected from 4 (exposed) and 2 (control) independently perfused placentae. **p* < 0.05 was considered statistically significant as analyzed by paired *t*-test. Abbreviations: DEPs: diesel exhaust particles; SD: standard deviation.

*Calculation of DEP exposure concentration from real-life conditions:* The DEP exposure level calculations were based on two approaches. As described in the main manuscript, the first approach is based on the previously determined average placental particle load of 2.09 x 10^4^ particles per mm^3^ in human placental tissue at term from mothers exposed to an average BC concentration ranging between 1.70-2.42 μg/m^3^ during pregnancy [[1]](https://paperpile.com/c/tT1PA1/3JGmQ). The second approach is based on the extrapolation from expected ambient exposure levels and the expected dose in the circulation for a 24 h exposure. Assuming a relatively high exposure of 25 µg/m^3^, a blood concentration of 450 ng could be achieved after 24 h of exposure with a daily inhaled air volume of 20 m^3^, a deposited fraction of 30% in the lungs, and a lung translocation of 0.3% [[2–4]](https://paperpile.com/c/cN8z0k/eXMvj+hBVTv+RePUb). Therefore, the highest possible particle load at the placental barrier (assuming 100% uptake) would be 450 ng for a 24 h exposure or 122 µg for a 9-month pregnancy exposure. Both approaches suggest a total particle exposure of 122-353 µg of particles per placenta over a 9-month exposure period (not adjusted for differences in the placental structure and perfusion conditions accompanied by different stages of pregnancy). For a single cotyledon (assuming an average of 20 cotyledons per placenta), 6.10-17.7 µg of particles could be expected. Taking a precautionary approach by choosing the highest concentration and including a 3x safety margin, experiments were performed with an applied concentration of 0.45 μg/mL (3 x 17.7 µg / 120 mL PM) in the maternal circulation for the perfusion studies. The safety factor of 3 was based on Ganguly et al. [[5]](https://paperpile.com/c/cN8z0k/ApEMu) and used to cover a worst-case scenario due to e.g., possible non-specific adsorption to the perfusion system (as previously observed for TiO_2_ [[6]](https://paperpile.com/c/cN8z0k/NDeAQ) and carboxylated gold [[7]](https://paperpile.com/c/cN8z0k/wbFQJ) nanoparticles), diffusion to adjacent non-perfused cotyledons (as reported for Benzo[α]pyrene [[8,9]](https://paperpile.com/c/cN8z0k/lymSC+J16wd) or incomplete tissue uptake of the applied particles.

*Adsorption of DEPs in the ex vivo perfusion device:* To determine a potential loss of freely diffusing DEPs to the perfusion system components, perfusion in the closed maternal compartment without placental tissue was performed. Therefore, the cannulas of the maternal device were placed into a falcon instead of being inserted into the placental tissue. The same DEP concentration (0.45 µg/mL) and volume (120 mL) were used in the *ex vivo* perfusion studies. At each time point
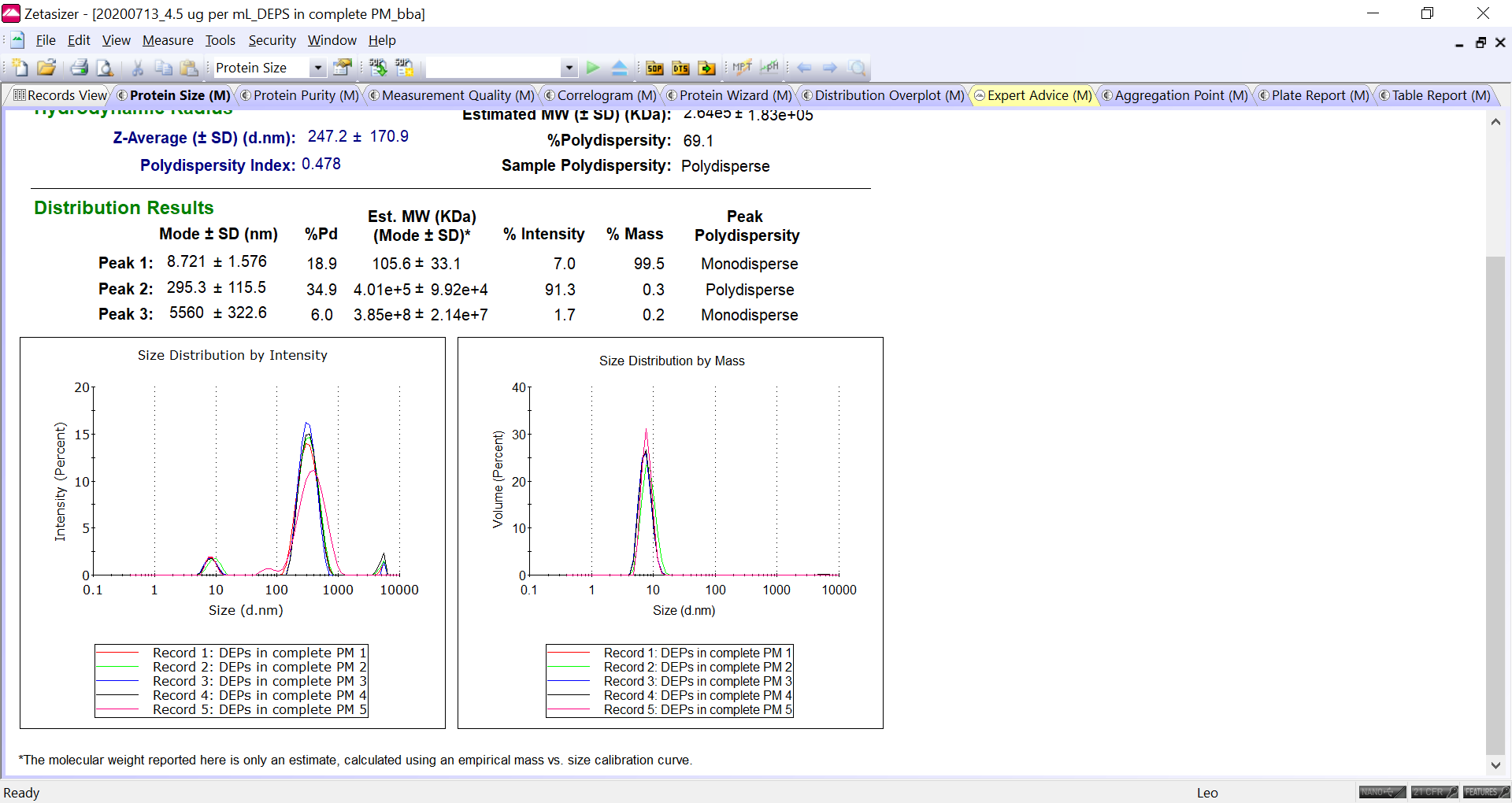
(0, 1, 3, and 6 h), 2 mL aliquots were collected and stored at -20 °C for further analysis.

**Figure S1. Size distribution by intensity of DEPs (0.45 µg/mL) in PM.** Peak 1: 8.7 ± 1.6 nm, Peak 2: 295.3 ± 115.5 nm, Peak 3: 5560.0 ± 322.6 nm, data are shown as Mode ± SD. Abbreviations: DEP: diesel exhaust particle; PM: perfusion medium; SD: standard deviation.


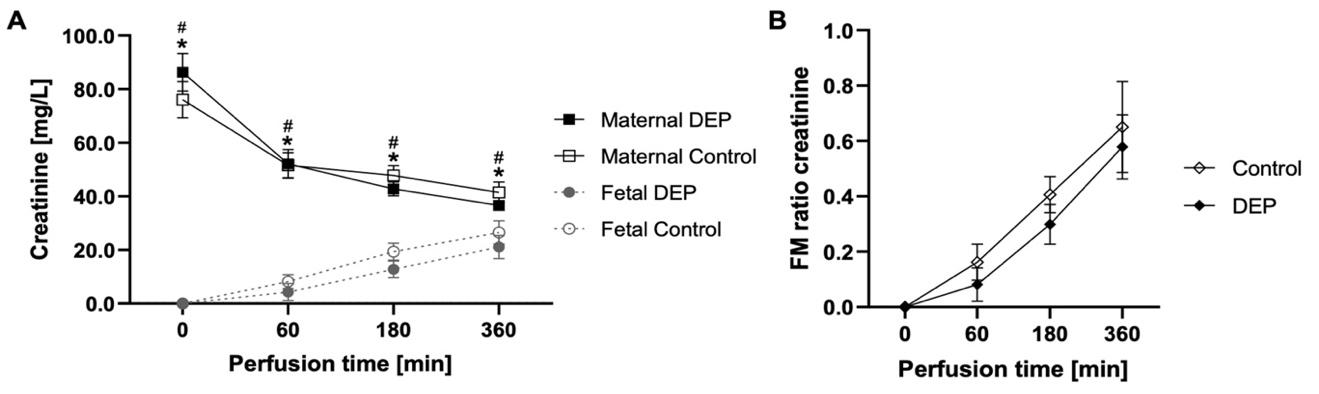
**Figure S2. Perfusion profiles and FM ratio of the reference compound creatinine.** Creatinine (100 mg/L) is added to the maternal circulation in independently perfused placentae from six different mothers (two placentae perfused with medium without DEPs to obtain baseline data (control) and four placentae perfused with medium containing DEPs). The concentration of the reference compound is measured from the maternal and fetal perfusates by a blood gas analyzer at several time points during 6 h of perfusion. Data represent the mean (SD) of two (control perfusions) and four (DEP perfusions) independently perfused placentae. *p* < 0.05 is considered statistically significant (* and ^#^ denote differences between maternal and fetal concentrations in control and DEP perfusions, respectively) as analyzed by one-way ANOVA with Tukey's multiple comparison correction. Abbreviations: ANOVA: analysis of variance; DEP: diesel exhaust particle; FM: fetal-**
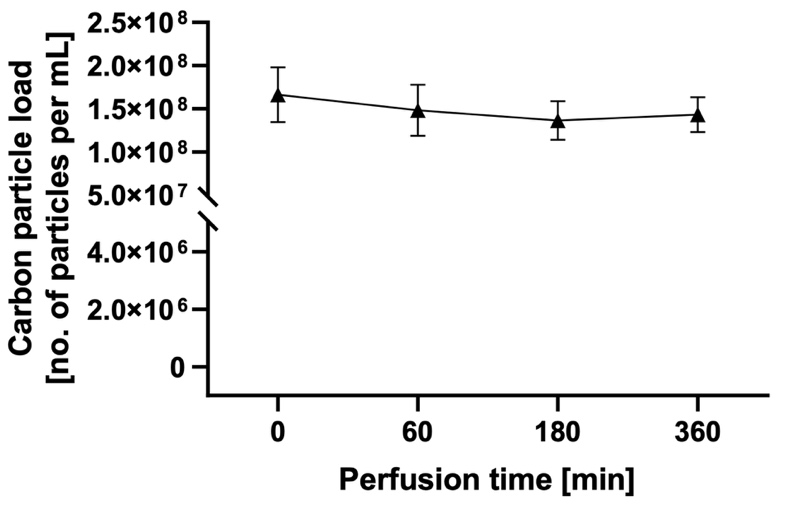
**maternal; SD: standard deviation.

**Figure S3. DEP absorbance to the perfusion system components.** The graph shows the carbon particle concentration in the perfusion medium over time when added to the maternal compartment of the *ex vivo* perfusion device (without placental tissue). 0.45 µg/mL DEPs were initially applied. Data are shown as the mean (SD) of three technical replicates. Abbreviation: DEP: diesel exhaust particle; SD: standard deviation.


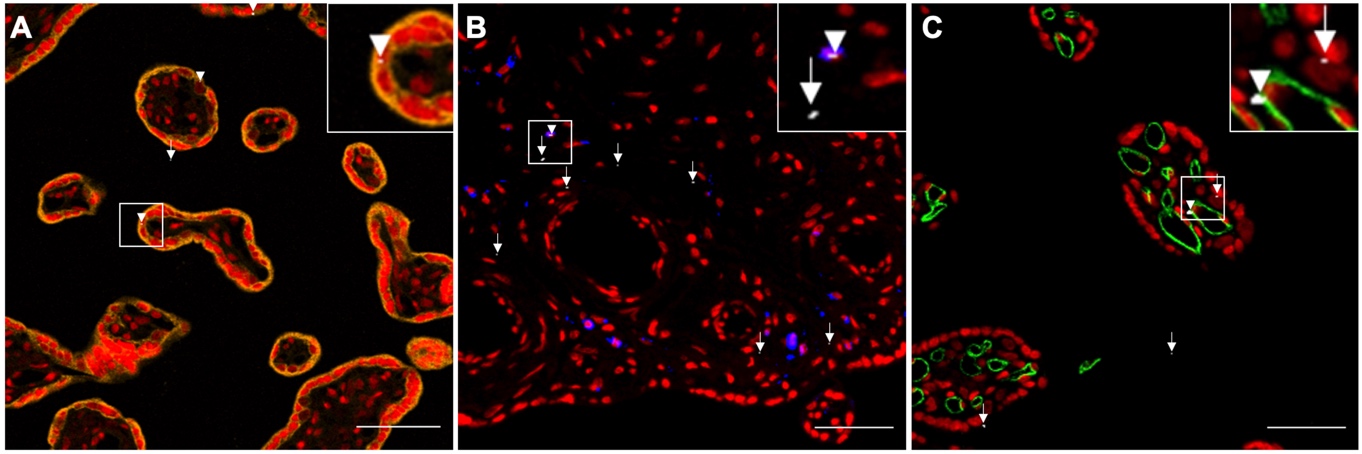


**Figure S4. Localization of carbon particles in placental villous tissue.** Images of placental tissue sections after 6 h of perfusion with empty PM. Trophoblast cells are stained with anti-cytokeratin (AE1/AE3, orange) (**A**), placental macrophages with anti-CD68 (blue) (**B**) and endothelial cells with anti-CD31 (green) (**C**). Syto 61 Red (red) was used as a nuclear counterstain (**A-C**). The carbon particles are imaged under femtosecond pulsed illumination (white; arrowheads and arrows indicate carbon particles colocalized or not with the stained cell type, respectively). Presented images are representative of all investigated samples. Scale bars: 100 µm. Abbreviation: PM: perfusion medium.

**References**

[1. Bové H, Bongaerts E, Slenders E, Bijnens EM, Saenen ND, Gyselaers W, et al. Ambient black carbon particles reach the fetal side of human placenta. Nat Commun. 2019;10:171.](http://paperpile.com/b/cN8z0k/XFbMI)

[2. Kreyling WG, Holzwarth U, Haberl N, Kozempel J, Wenk A, Hirn S, et al. Quantitative biokinetics of titanium dioxide nanoparticles after intratracheal instillation in rats: Part 3. Nanotoxicology. 2017;11:454–64.](http://paperpile.com/b/cN8z0k/eXMvj)

[3. Geiser M, Kreyling WG. Deposition and biokinetics of inhaled nanoparticles. Part Fibre Toxicol. 2010;7:2.](http://paperpile.com/b/cN8z0k/hBVTv)

[4. Kreyling WG, Hirn S, Möller W, Schleh C, Wenk A, Celik G, et al. Air-blood barrier translocation of tracheally instilled gold nanoparticles inversely depends on particle size. ACS Nano. 2014;8:222–33.](http://paperpile.com/b/cN8z0k/RePUb)

[5. Ganguly K, Ettehadieh D, Upadhyay S, Takenaka S, Adler T, Karg E, et al. Early pulmonary response is critical for extra-pulmonary carbon nanoparticle mediated effects: comparison of inhalation versus intra-arterial infusion exposures in mice. Part Fibre Toxicol. 2017;14:19.](http://paperpile.com/b/cN8z0k/ApEMu)

[6. Aengenheister L, Dugershaw BB, Manser P, Wichser A, Schoenenberger R, Wick P, et al. Investigating the accumulation and translocation of titanium dioxide nanoparticles with different surface modifications in static and dynamic human placental transfer models. Eur J Pharm Biopharm. 2019;142:488–97.](http://paperpile.com/b/cN8z0k/NDeAQ)

[7. Aengenheister L, Dietrich D, Sadeghpour A, Manser P, Diener L, Wichser A, et al. Gold nanoparticle distribution in advanced in vitro and ex vivo human placental barrier models. J Nanobiotechnology. 2018;16:79.](http://paperpile.com/b/cN8z0k/wbFQJ)

[8. Mathiesen L, Rytting E, Mose T, Knudsen LE. Transport of benzo[alpha]pyrene in the dually perfused human placenta perfusion model: effect of albumin in the perfusion medium. Basic Clin Pharmacol Toxicol. 2009;105:181–7.](http://paperpile.com/b/cN8z0k/lymSC)

[9. Mose T, Kjaerstad MB, Mathiesen L, Nielsen JB, Edelfors S, Knudsen LE. Placental passage of benzoic acid, caffeine, and glyphosate in an ex vivo human perfusion system. J Toxicol Environ Health A. 2008;71:984–91.](http://paperpile.com/b/cN8z0k/J16wd)
